# Multilayer cyberattacks identification and classification using machine learning in internet of blockchain (IoBC)-based energy networks

**DOI:** 10.1016/j.dib.2024.110461

**Published:** 2024-05-03

**Authors:** Muhammad Faheem, Mahmoud Ahmad Al-Khasawneh

**Affiliations:** a Department of Computing Science, School of Technology & Innovations, University of Vaasa, Vaasa 65200, Finland; b Vaasa Energy Business and Innovation Centre (VEBIC), University of Vaasa, Vaasa 65200, Finland; c School of Digital Economy, University of Vaasa, Vaasa 65200, Finland; d School of Computing, Skyline University College, University City Sharjah, Sharjah 1797, United Arab Emirates

**Keywords:** Cybersecurity, Blockchain, Deep learning, Long short-term memory, Renewable energy, Smart grid

## Abstract

The world's need for energy is rising due to factors like population growth, economic expansion, and technological breakthroughs. However, there are major consequences when gas and coal are burnt to meet this surge in energy needs. Although these fossil fuels are still essential for meeting energy demands, their combustion releases a large amount of carbon dioxide and other pollutants into the atmosphere. This significantly jeopardizes community health in addition to exacerbating climate change, thus it is essential need to move swiftly to incorporate renewable energy sources by employing advanced information and communication technologies. However, this change brings up several security issues emphasizing the need for innovative cyber threats detection and prevention solutions. Consequently, this study presents bigdata sets obtained from the solar and wind powered distributed energy systems through the blockchain-based energy networks in the smart grid (SG). A hybrid machine learning (HML) model that combines both the Deep Learning (DL) and Long-Short-Term-Memory (LSTM) models characteristics is developed and applied to identify the unique patterns of Denial of Service (DoS) and Distributed Denial of Service (DDoS) cyberattacks in the power generation, transmission, and distribution processes. The presented big datasets are essential and significantly helps in identifying and classifying cyberattacks, leading to predicting the accurate energy systems behavior in the SG.

Specifications Table

**Table:** 

Subject	Computer Science: Computer Communication Networks, Distributed Energy Systems, Renewable Power Systems.
Specific subject area	Cybersecurity, Machine learning.
Data format	Raw and Analyzed
Type of data	Tables, Graphs, Figures
Data collection	Big datasets were collected using various kinds of IoT-enabled Industrial Wireless Sensor Networks (IWSNs) (temperature, pressure, angular motion, smoke, wind direction, etc.) operating at 2.4GHz, specifically employing IEEE physical layer standards 802.11 and 802.15.4, with a data transmission speed of 250 kbits/s and an effective communication range of 7 meters. The Intelligent Electronic Devices (IEDs) operating on IEC 61850, responsible for measuring frequency, current, voltage, etc., were interfaced with the sensors during deployment for monitoring events in a solar park and wind farm. Both the IEDs and IoT-enabled sensor nodes adeptly collected various types of event information from wind turbines and solar panel systems within a static topology. This information was then transmitted to the sink via a multi-hop message passing manner. In turn, the sink forwards the collected data in real-time to the smart grid control centre, utilizing 5G wireless communication technology for further investigations. In the established network, we assumed that adversary A launches a set of DoS and DDoS attacks to manipulate the energy and power systems data with the intention of gaining control over the DERs in the SG. A set of 250 nodes, each quipped with a unique were involved in the data collection process in both solar park and wind farm. The entire setup, including the wind farm, solar park, as well as the blockchain-based communication infrastructure and machine learning model components, were modeled using the real-time simulator RTDS/OPAL-RT and Fedora 32. In addition, Java and C++ programming tools have been employed for modelling the HML model in the SG. Measurement frequencies were configured for real-time acquisition, set at intervals of 15 minutes.
Data source location	Institution: University of Vaasa City/Town/Region: Palosaari, 65200, Vaasa. Country: Finland. Latitude and longitude for collected samples/datasets: 63°06′13.6"N 21°35′36.4"E.
Data accessibility	Datasets are available at the Mendeley Data repository. Direct URL to data: https://data.mendeley.com/datasets/zc9z7m7gcd/1 Doi: 10.17632/zc9z7m7gcd.1
Related research paper	Datasets have not been published previously and belongs to our research work [1].

## 1 Value of the Data

• The data provides insights into cyberattacks, helping in the development of predictive models that can anticipate future threats and vulnerabilities in energy and power systems.
• Data analysis helps in customizing security protocols and measures tailored to specific threats and vulnerabilities in distributed energy systems, enhancing overall system security.
• Data contributes to a better understanding of the current security posture of the energy and power systems, aiding in strategic decision-making and resource allocation for cybersecurity.
• Cybersecurity and smart grid agencies, along with other stakeholders, can leverage these datasets to develop a more intelligent and resilient data exchange network. This forward-thinking strategy will help in identifying and mitigating different types of cyberattacks, ensuring the protection of the confidentiality of employees, companies, and clients.

## 2 Background

The increasing global demand for energy is met primarily by fossil fuels like coal and gas, leading to significant emissions of carbon dioxide and pollutants [2,3]. This poses health risks and intensifies the problem of climate change. To address these challenges, it's crucial to incorporate green energy sources like hydropower, wind, and solar power in the smart grid by using blockchain-based advanced information and communication technologies [[4], [5], [6]]. Table 1 highlights various characteristics of blockchain technology in the smart grid [[7], [8], [9]]. However, there are several critical cybersecurity issues, which brings unique challenges to the reliability, stability, and resilience of the smart grid [10,11]. In recent years, the scientific community acknowledges the importance of machine learning technology since it plays a crucial part in predicting current and future behavior in various industrial applications. In smart grid, machine learning could be beneficial in terms of analyzing unique data patterns to identify and classify the different cyberattacks behavior to improve the behavior of energy, [[12], [13], [14]]. Table 2 highlights various types of machine learning algorithms, their strength, weaknesses, and potential applications [[15], [16], [17]]. Consequently, this study presents cybersecurity datasets collected from wind turbines and solar panels in energy systems, which were not fully explored previously systems [[18], [19], [20]]. These datasets offer new opportunities for analysis and visualization, enhancing understanding of a cybersecurity framework's effectiveness in energy and power systems. The comprehensive data contributes to evaluating the cybersecurity framework's potential and limitations, encouraging further research and innovations in the smart grid.

**Table 1:** Various blockchain techniques in smart grid.

Metrics	Bitcoin	Ethereum	Aptos	Solana	Palkadot	Avalanche	ADA	Ripple	Algorand
**Type of blockchain**	Layer 1	Layer 1	Layer 1	Layer 1	Layer 1	Layer 1	Layer 1	Layer 1	Layer 1
**Architecture**	Pub/Pvt	Pub/Pvt	Pub/Pvt	Pub/Pvt	Pub/Pvt	Pub/Pvt	Pub/Pvt	Pub/Pvt	Pub/Pvt
**Consensus mechanism**	PoW	PoS	PoS	PoS and PoH	PoS	Avax Consensus	Ouroboros (PoS)	RPCA	PoS
**Maximum transaction/s**	7+tps	45+tps	160,000	5,000+tps	1,500+tps	10,000+tps	250+tps	1500+tps	1000+tps
**Hash Function**	SHA-256	Keccak-256	SHA-256	SHA-256	Blake2b	secp256k1	BLAKE2b	SHA-512	SHA-512/256
**Time-To-Finality**	60 minutes	6 minutes	<10 second	400ms	6 minutes	< 2 second	∼20 minutes	4-5 second	< 5 sec
**Number of Validators**	Open	Open	Open	1700+	<200 nodes	Open	Open	>150 +	Open
**Safety Threshold**	51%	51%	33%	66%	33%	80%	-	-	-
**Programming language**	C++	Solidity	Move	Rust, C, C++, Python	Rust to JavaScript	Go, JavaScript, Python, Vue	Plutus, Haskell, Marlowe	C++	Go, Teal, Pyteal, Phthon
**Model type**	Dec	Dec	-	-	Dec	-	Dec	Cen	-
**Smart Contracts**	No (Script language)	Yes (EVM)	-	Yes (Sol EVM)	Substrate, EVM	Avax C-chain	Yes	No (XRP Ledger)	-
**Features**	-	DeFi, NFTs	-	Web3	Relay chain, Para chains	Quick finality, Set of subsets	DeFi,	-	-
**Complexity**	Medium	High	Medium to High	High	-	O(kn)	High	Medium	Medium to High
**Latency**	High	Moderate	Low	Low	Moderate	Low	Moderate	Low	Low
**scalability**	Low	Moderate	Moderate	High	High	High	High	Moderate	High
**Energy Efficiency**	No	No	No	Yes	Yes	Yes	Yes	Yes	Yes
**Privacy Features**	Limited	Limited	Limited	Limited	Limited	Limited	Enhanced	Limited	Limited

**Table 2:** Machine learning techniques with their applications, strengths, and weaknesses in smart grid.

Supervised Learning				
Category/Model	Description	Applications	Strengths	Weaknesses
**Supervised Learning**	Models that learn from labeled training data to make predictions	• Classification • Regression • Image Recognition	• Direct feedback • Easier to understand and implement Wide range of applications	• Requires large amounts of labeled data • Prone to overfitting if not managed properly • May not generalize well to unseen data
**Classification of Supervised Learning**				
**Decision Trees**	Tree-like model of decisions and their possible consequences	• Customer segmentation • Fraud detection	• Easy to interpret • Handles both types of data	• Prone to overfitting • Sensitive to changes in data • Can create complex trees that do not generalize well
**Random Forest**	An ensemble method using multiple decision trees to improve classification or regression accuracy	• Fraud detection in banking • Medical diagnosis • Stock market prediction	• Handles overfitting better than decision trees • Good performance • Handles categorical and numerical features well	• Can be complex and require more computational resources • Less interpretable than decision trees
**SVM**	A supervised learning model that finds the best hyperplane to separate different classes in the feature space	• Face detection • Text categorization • Classification of images	• Works well with clear margin of separation • versatile (different kernel functions) • Effective in high dimensional spaces	• Requires feature scaling; • Not suitable for large datasets • Sensitive to kernel choice
**Neural Networks**	Models inspired by the human brain, consisting of neuron layers that process inputs to make complex decisions	• Speech recognition • Image recognition, • Natural Language Processing	• Highly flexible and complex model • Non-linear relationships • Performs well on large datasets	• Requires a lot of data • Computationally expensive • Prone to overfitting & less interpretable
**Naïve Bayes**	It is a simple probabilistic classifier that uses Bayes' theorem and strong (naive) independence assumptions between features	• Spam filtering • Sentiment analysis • Document classification	• Simple and easy to implement • No training phases • Versatile (useful for sorting and regression)	• Difficulty in capturing relationships between features • Low performance with high-dimensional data sets • Strong independence assumption, which is unrealistic
**KNN**	A non-parametric method used for classification and regression by analyzing the closest k data points	• Recommender systems • Image classification • Pattern recognition	• Simplicity and ease of implementation • Adaptability and no assumptions on data • Versatility for different tasks	• Slow on large datasets • Sensitive to irrelevant features and the scale of data • Requires feature scaling
**Regression**				
**Linear Regression**	Predicts a continuous outcome based on one or more variables	• Predicting sales • Real estate pricing	• Simple and interpretable • Efficient	• Assumes a linear relationship Sensitive to outliers • Can't model complex relationships
**Polynomial Regression**	Identify a nonlinear relationship between the value of x and the corresponding conditional mean of y.	• Modeling nonlinear relationship • Economic growth • Behavioural analysis	• Can model complex relationships more accurately than linear regression • Flexible approach to modeling curves in data	• Prone to overfitting, especially with high-degree polynomials • Requires careful selection of the degree of the polynomial • High computational cost with increased degree of polynomial
**Ridge/Lasso Regression**	Both are types of regularized linear regression that add a penalty term to the cost function.	• Feature selection • Predicting in clinical trials • Financial forecasting	• Ridge reduces model complexity and prevents overfitting by shrinking coefficients • Lasso effectively performing feature selection	• Ridge does not perform feature selection • Lasso can unpredictably behave in the presence of highly correlated features
**Logistic Regression**	Used for binary classification problems	• Medical diagnosis • Email spam detection	• Provides probability scores • Good for binary outcomes	• Assumes linearity between dependent and independent variables • Limited to binary or ordinal outcomes
**Unsupervised Learning**				
**Unsupervised Learning**	Models that identify patterns in unlabelled data	• Clustering, generative model • Dimensionality Reduction • Anomaly detection	• No need for labeled data • Good for exploratory analysis	• More challenging to validate results • No definitive way to predict outcomes • Less accuracy compared to supervised models
**K-Means Clustering**	Partitions data into k distinct clusters based on distance to the centroid of the cluster	• Market segmentation • Document clustering	• Simple and efficient • Easy to interpret	• Inefficient for capturing clusters of unlike shapes and sizes • Inability to identify non-convex clusters • Outliers can heavily influence the calculation of centroids
**Hierarchical Clustering**	A method of cluster analysis which seeks to build a hierarchy of clusters	• Gene sequence analysis • Social network analysis • Market segmentation	• Not require a pre-specified number of clusters • Easy to interpret and visualize • Can capture complex structures	• Scalability issues with large datasets • Sensitive to noise and outliers • Finding the optimal number of clusters can be subjective
**DBSCAN**	Density-Based Spatial Clustering (DBCAN) identifies clusters of high density from noise	• Anomaly detection • Geospatial data analysis • Image segmentation	• Can find arbitrarily shaped clusters • Robust to outliers • Not require specifying the number of clusters	• Sensitive to parameter settings (eps and minPts) • Struggles with varying density clusters • Performance can degrade in high-dimensional space
**Mean-Shift**	A non-parametric clustering that iterates through candidate centroids until convergence, via feature space analysis	• Image processing • Object tracking • Data analysis	• Finding clusters regardless the number • Robust to outliers • Capable of handling non-linear feature spaces	• Computationally expensive, especially with large datasets • Performance depends on bandwidth parameter • May converge to local maxima
**Apriori**	It iterates through datasets to find subsets that frequently occur together	• Market basket analysis • Cross-marketing strategies • Catalog design	• Easy to understand and implement • Can be parallelized to improve efficiency • Effective in large datasets	• Slow due to the exponential growth of candidate sets • Memory-intensive • Requires multiple scans of the database
**Eclat**	Equivalent Class Clustering and bottom-up Lattice Traversal is used for mining frequent item sets	• Market basket analysis • Association rule mining • Recommendation systems	• Faster than Apriori due to reduced overhead • Uses set intersection to count supports, improving efficiency & scalable to large datasets	• Memory usage can be high for dense datasets • Performance can degrade with very large datasets • Still requires multiple scans of the dataset
**PCA**	Cconvert a set of annotations of correlated variables into a set of values of linearly uncorrelated variables	• Feature reduction • Data visualization	• Reduces complexity • Removes correlated features	• Assumes linear relationships between variables • May not capture complex structures compared to nonlinear • Key components are less interpretable than original features
**t-SNE**	t-SNE minimizes pairwise similarities of the input data and measures pairwise similarities	• Data visualization • Exploratory data analysis • Clustering identification	• Intuitive visualizations of high-dimensional data • Revealing clusters and structures at unlike scales • Useful for exploratory data analysis	• Computationally intensive, especially for large datasets • Results can vary significantly based on hyperparameters • Not suitable for dimensionality reduction
**Autoencoder**	Used to learn efficient codings of unlabeled data	• Feature learning • Anomaly detection • Denoising images	• Learning nonlinear and complex data structures • Useful for unsupervised learning of data coding • Used for denoising and anomaly detection	• Risk of learning the identity function (overfitting) • Architecture and hyperparameters is critical for performance • Less interpretable than linear methods like PCA
**Semi-supervised Learning and Reinforcement Learning**				
**Reinforcement Learning**	Learning what actions to take in an environment to maximize a reward	• Game AI • Robotics	• Learns by interacting with the environment • Adaptable to new scenarios	• Requires a well-defined reward system • Can be unstable or converge slowly
**Q-Learning**	A model-free reinforcement learning algorithm	• Board games • Navigation systems	• Off-policy learning • Finds optimal action-selection policy	• Can exhibit instability and convergence issues • Scalability with High-Dimensional State Spaces • Lack of Exploration Strategy
**DQN**	The model learns to achieve a policy that maximizes the expected reward by interacting with the environment	• Video game playing • Robotics for control tasks • Decision-making in finance	• Solve high-dimensional observation spaces • Stable learning due to replay and fixed Q-targets	• Struggles with high-dimensional action spaces • Limited applicability to continuous domains • Can overestimate Q-values, leading to suboptimal policies
**Policy Gradients**	They work by estimating the gradients of expected reward with policy parameters to maximize the reward	• Robotic manipulation and locomotion, Game playing • Optimization problems	• Suited for high-dimensional action spaces • Can learn stochastic policies • Efficient learning due to directly optimization.	• Slow convergence due to high variance in gradient estimate • May converge to local optima, failing to find the best possible policy
**Actor-Critic Methods**	Actor-Critic methods combine the ideas of policy gradient methods (actor) and value function approximation (critic)	• Decision-making in robotics • Autonomous vehicles • Gaming, healthcare, energy	• High-dimensional and continuous action space • Faster convergence than policy gradient • Policy gradient and value functions policy	• Requires careful design and balance between the actor and critic updates to avoid instability • Added complexity due to actor and critic models
**Deep Learning (Subcategory of Neural Networks)**				
**Deep Learning**	Involves neural networks with many layers	• Image and speech recognition • Natural Language Processing	• Handles large and complex data • High accuracy in tasks like image recognition	• Requires substantial computational resources • Needs large amounts of data • Complex to design and tune
**CNN**	Particularly good for processing pixel data	• Image and video recognition • Image classification	• State-of-the-art for image tasks • Efficient feature learning	• High computational cost • Requires large amounts of labeled data • Primarily suitable for image-related tasks
**LSTM**	A type of RNN effective in learning order dependence in sequence prediction problems	• Time series prediction • Text generation	• Handles long-term dependencies • Good for sequential data	• Computationally expensive • Prone to overfitting on smaller datasets • Requires careful tuning of parameters
**RNNs**	RNNs are capable of handling data of varying lengths by maintaining a state that is passed from one step to the next	• Language modeling • Speech recognition • Time series analysis	• Good at capturing sequential data patterns • Flexible in processing data of different lengths • Suitable for various sequence prediction tasks	• Difficulty in learning long-term dependencies • Computationally intensive for long sequences • Prone to overfitting on smaller datasets
**GANs**	GANs learns to produce data resembling the training set, and learns to distinguish between real and generated data	• Image generation • Creating realistic artworks • Data augmentation	• Capable of generating high-quality, realistic images or data • Useful in unsupervised learning scenarios	• Training stability issues • Mode collapse with limited varieties of samples • Lack of considering the traditional metrics.
**Transformer Models (e.g., BERT, GPT)**	They process input data in parallel to improve the limitations of handling sequential data	• Language translation • Content generation • Sentiment analysis	• Efficient at processing large sequences in parallel and due to contextual relationships • Scalable with increasing data and model size	• High computational and memory requirements for training • Prone to generating biased or nonsensical text outputs • Requires substantial amounts of data for training effectively
**Fusion Techniques**				
**Ensemble Methods**	Techniques that combine several machine learning models to improve performance	• Classification • Regression • Prediction	• Improved accuracy • Reduces model biases • Balances variance and bias	• Computationally intensive • More complex to implement and tune • Risk of increased bias if base models are biased
**Bagging (Bootstrap Aggregating)**	Creates multiple models on subsets of data, averaging their predictions	• Random forests • Regression models	• Reduces overfitting • Works well with high variance models	• Models are independently constructed, which can ignore interactions • Can be less effective if individual models are biased
**Boosting**	Sequentially builds models, each correcting the errors of the previous one	• AdaBoost • Gradient boosting	• Often provides higher accuracy • Good for reducing bias and variance	• More sensitive to overfitting with noisy data • Computationally more intensive • Requires careful tuning of parameters
**Stacking**	Combines the predictions of multiple models using another model	• Classification and regression tasks	• Can yield higher accuracy than any single model • Flexibility in model choice	• Complex to implement correctly • Risk of overfitting on the meta-model • Choosing the right combination of models is crucial
**Feature Fusion**	Combines different types of features (e.g., textual, visual) to improve model performance	• Multimodal data analysis • Image and text analysis	• Comprehensive data representation • Can improve performance significantly	• Increases the dimensionality of the data • Risk of introducing irrelevant or redundant features • Requires careful feature selection and pre-processing
**Early Fusion**	Combines features at the beginning of the process	• Sensor data integration • Multimedia analysis	• Simplified model architecture • Direct interaction of features	• Sensitivity to Noise and Outliers • Difficulty in Model Interpretation • Less scalable, especially with large datasets.
**Late Fusion (Decision Fusion)**	Combines decisions or outputs from multiple models	• Medical diagnosis • Multimodal Sentiment Analysis	• Maintains model independence Flexibility in using different models	• Decision rules can be complex to determine • Requires well-calibrated and diverse models • Potentially more sensitive to errors in individual models
**Data Fusion**	Integrates data from multiple sources for a more comprehensive view	• Healthcare Monitoring • Environmental Sensing	• Richer insights from diverse data • Can improve model robustness	• Combining multiple data sources can lead to a high-dimensional space, complexity and computational cost
**Model Fusion**	Combines multiple models or algorithms to create a more robust system	• Robotic Control Systems • Complex Prediction Tasks	• Combines strengths of various algorithms • Can improve performance and accuracy	• Using multiple models can increase the risk of overfitting, particularly if the models are highly correlated

## 3 Data Description

**Fig. 1:**
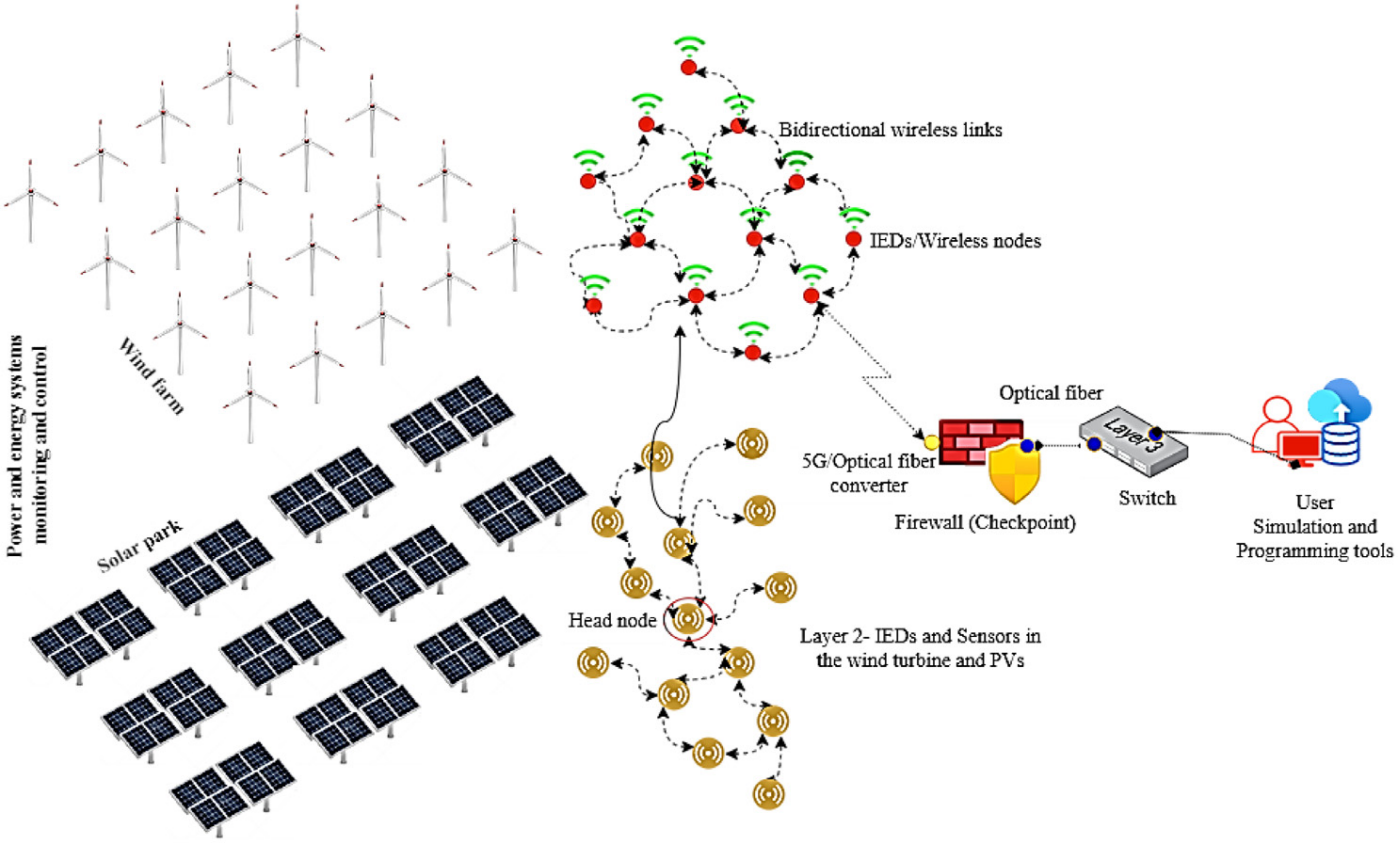
Wind and solar powered DERs in SG [1].

This study presents datasets obtained from the deployment of IoT-enabled advanced Solana blockchain-based IWSNs and IEDs deployed for the purpose of monitoring and controlling events across spatially dispersed solar panels and wind turbines in the SG. The existing datasets offer an in-depth examination of various cyberattack modalities, delineating their occurrence rates, and elucidating the strategies employed by malefactors targeting critical energy and power infrastructures. The data gathering mechanism involved statically deployed nodes tasked with the continuous monitoring and recording of a wide array of environmental and operational parameters, including, wind direction, velocity, ambient temperature, humidity levels, smoke detection, proximity, motion, structural integrity (cracks), electrical current, voltage, and frequency metrics. As depicted in Fig. 1, the data acquisition process involved the collection and transmission of energy and power systems data from the solar park and wind farm directly to the remote data center, leveraging a hybrid communication infrastructure that combines 5G and optical fiber technologies in the SG. Subsequently, the collected data is securely stored on an MS SQL server situated in the SG. To ensure the datasets applicability and facilitate their future use, they have been accurately structured in the .CSV format (available at: https://data.mendeley.com/datasets/zc9z7m7gcd/1).

**Fig. 2:**
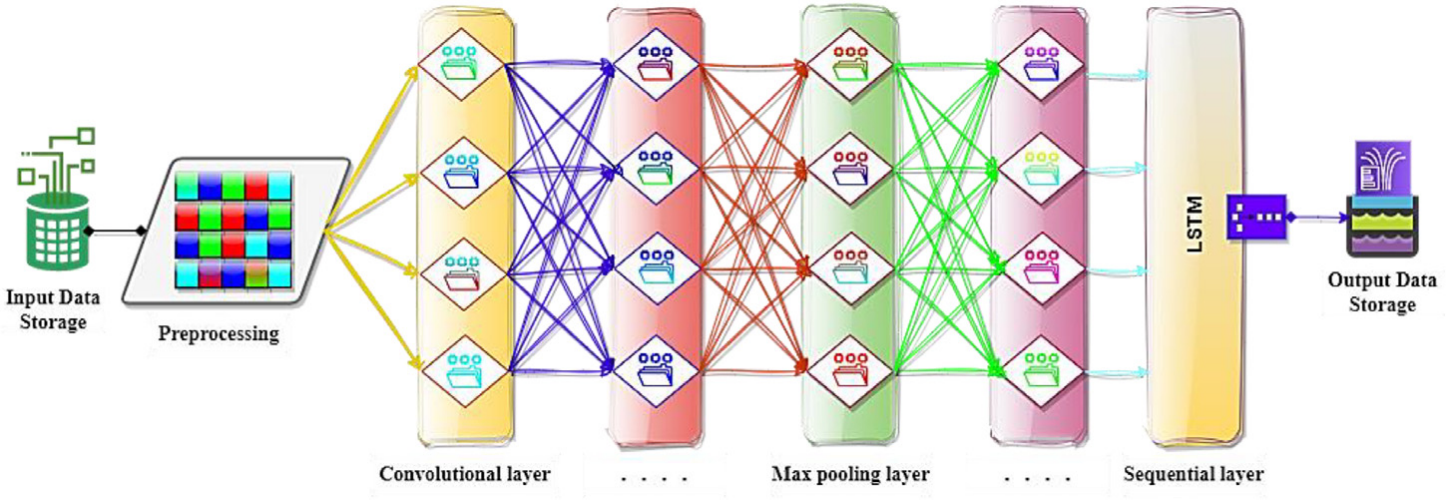
A hybrid machine learning model for DERs in SG [1].

Throughout the surveillance and management phases, the network was exposed to a series of DoS and DDoS cyberattacks, aimed at compromising data integrity, effectuating unauthorized data manipulation, and usurping identity verification of the energy and power systems, including the users and utilities. A DoS attack is a cyber-attack where the attacker seeks to make a power systems or energy network resource unavailable to its intended users or neighboring devices by temporarily or indefinitely disrupting services of a host connected to the internal and external network. This is achieved by overwhelming the target with a flood of requests or packets, causing the system to slow down or crash, thereby denying service to legitimate users and systems. The attack can be executed from a single internal or external internet connection, targeting one or more websites, servers, or other resources. Common methods include flooding the network to prevent legitimate network traffic, disrupting connections between two machines, preventing access to a service, or exhausting resources in a targeted device. Instead, a DDoS attack is similar to a DoS attack, but the attack originates from multiple, often thousands of internal or external sources. This makes it much harder to stop because blocking a single source doesn't sojourn the attacks. In the DDoS attack, a network of intelligent devices like computers (often part of a botnet) is used to flood the target with an overwhelming amount of traffic. This can include requests for connections, messages, or malformed packets, with the goal of exhausting the target's resources. DDoS attacks can be volumetric (increasing traffic to saturate the bandwidth), protocol attacks (targeting network layer protocols), or application layer attacks (targeting web applications with seemingly legitimate requests). DDoS attacks are generally more complex and difficult to mitigate than DoS attacks because they involve multiple distributed sources in distributed energy and power systems. Both types of attacks can cause significant damage to existing energy and power infrastructures by disrupting services, causing financial loss, and damaging reputations of the energy utilities. In order to identify these imperceptible cyber attacks, a hybrid machine learning model as shown in Fig. 2 is designed to perform the rigorous analysis of the collected big datasets to uncover their recurring patterns, highlighting their inherent vulnerabilities in the smart grid.

(1) $$\mathrm{Input}\;\left(D_{i}\right)=\sum_{i=1}^{n}D{\left|x\times y\right|}^{t_{j}}$$

(2) $$D_{i\left(t_{j}\right)}=\int_{i}^{n}DL{\left|x\times y\right|}^{t_{j}}+\int_{i}^{m}LSTM{\left|x\times y\right|}^{t_{j+1}}\;$$

(3) $$\mathrm{Output}\;\left(D_{i+1}\right)=\sum_{i=1}^{n}D{\left|x\times y\right|}^{t_{j+1}}\;$$

In the initial phase, data is collected from various devices through a blockchain-based communication network and stored in the input data storage at the data center as shown in Fig. 2. Following initial preprocessing, attributes and weights for each metric are accurately determined to assess changes in the original data values. The proposed HML model employs DL and LSTM techniques for analyzing big datasets to identify the precise patterns with their initial true values in the smart grid. The DL model utilizes CNN layers to process raw data, enabling the effortless identification of vital features without the need for manual intervention. The architecture of the CNN consists of convolutional layers, max pooling layers, and sequential layers. Convolutional layers are mainly responsible for feature extraction, while the max pooling layers contribute to minimizing the datasets dimensionality and increasing robustness. Sequential layers known as a layered structure, simplify the development of linearly connected neural networks, thereby enhancing the efficiency of recognizing patterns and structures of the energy systems datasets. LSTM, a specialized type of recurrent neural network, is proficient at handling sequences and understanding the long-term dependencies, making it ideal for time-series energy systems datasets in smart grid. It is proficient of analyzing temporal dynamics and learning from event sequences to either predict future outcomes or classify anomalies. The process starts with the preprocessing of energy and power systems data, during which relevant features are extracted using CNN technique. This extracted data is then fed into an LSTM model, where it undergoes analysis to assess temporal dependencies and sequential patterns. The LSTM model is specifically designed to refine the energy and power systems datasets, leveraging its advanced temporal analysis proficiencies to improve the relevance and accuracy of the data values significantly. This step is vital for processing the data with high precision and guaranteeing that it imitates the most pertinent information with highest accuracy. The combination of DL for spatial feature extraction and LSTM for modeling time-dependent aspects advances the detection of anomalies in complex datasets of energy systems. Finally, the updated information is stored in the output data storage as results, signifying the accomplishment of an advanced data processing cycle.

Eq. 1 shows the multidimensional input data $D_{i}$ in matrix |x×y| received from different energy systems in time $t_{j}$ in the smart grid. The data is collected from various kinds of sensors installed on different distributed energy and power systems, operating on blockchain-based communication network in the smart grid (as discussed in detail in experimental design, materials, and methods Section). Eq. 2 illustrates the DL method, which is applied on the received data matrix |x×y| in time $t_{j}$ and the output of this process is then forwarded to the LSTM model in time $t_{j+1}$. It significantly improves the capacity to identify anomalies within complex energy and power systems datasets in the smart grid. Lastly, Eq. 3 describes the output data obtained from both DL and LSTM model is stored in the data storage as output for observing the change in the original datasets in time $t_{j+1}$ in the smart grid.

**Fig. 3:**
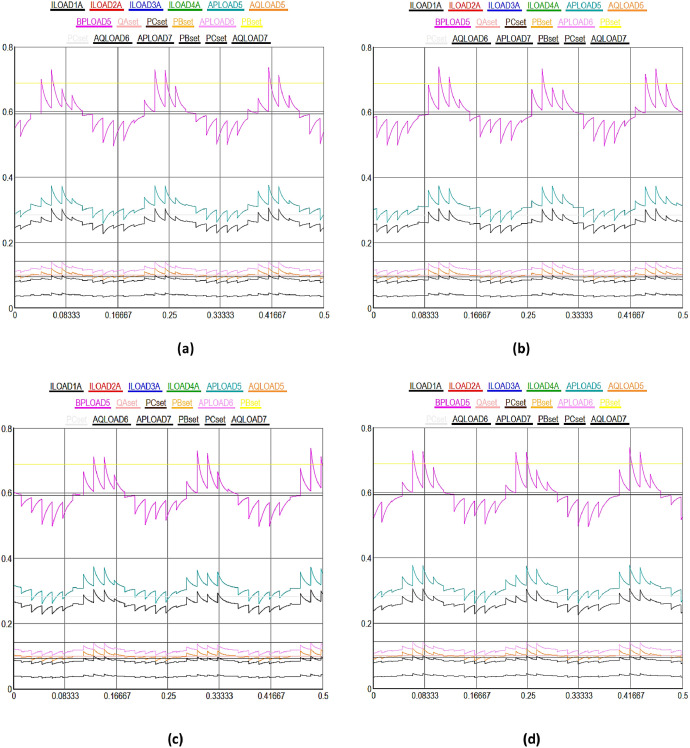

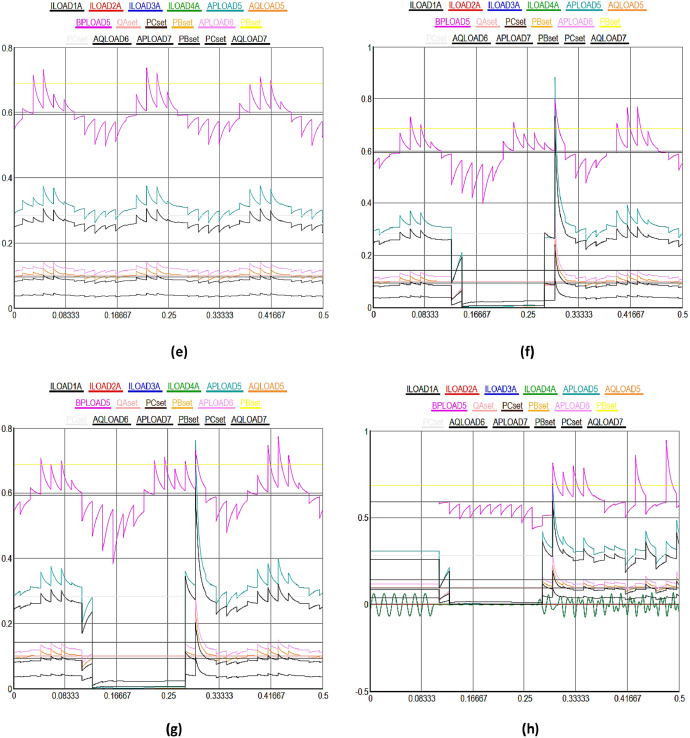

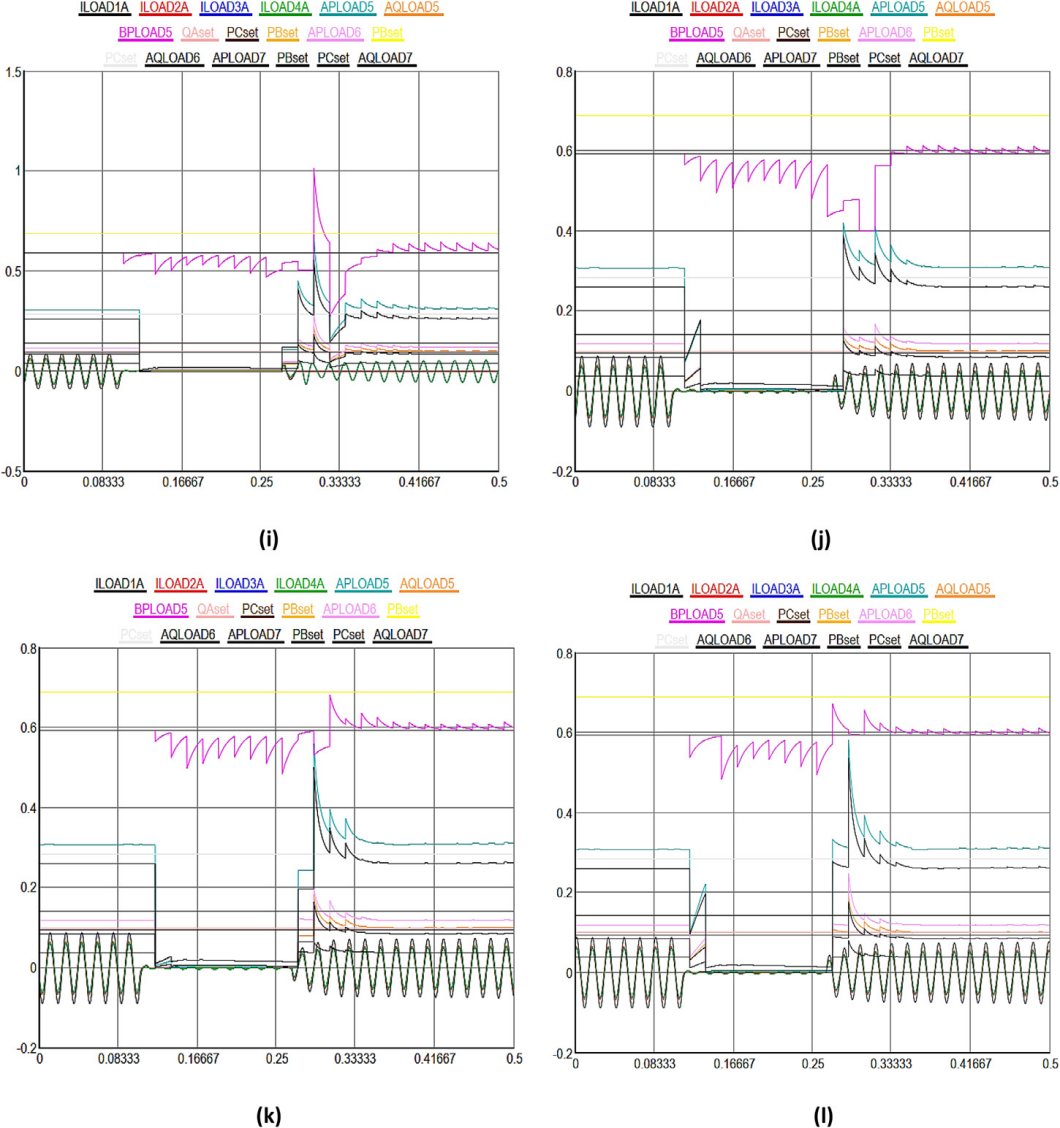

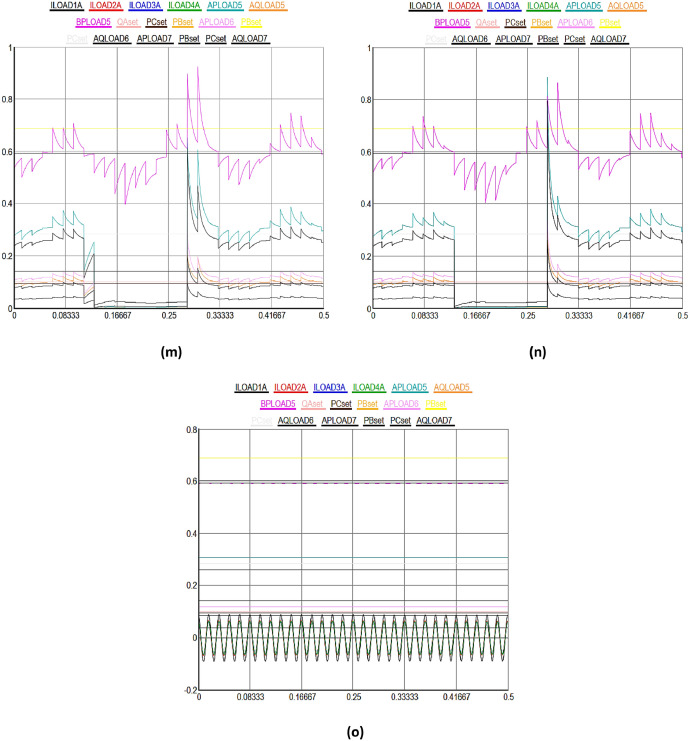
Multidimensional data collection using IEDs and sensors in DERs in SG.

Figs. 3 shows a detailed view of the multidimensional data collection process from a variety of IEDs and sensors in DERs in SG. In Fig. 3, X-axis highlights the upper most limit of the received data signal values set to 0.8%, while Y-axis shows the different time domain between 0 and 0.5sec in the SG. Figs. 3(a) to (e) illustrate normal data flow between different solar and wind powered system components in the smart grid. Fig. 3(a) highlights the various kinds of data signals received from geographically distributed wind turbines in a wind farm. Moving on to solar energy, Fig. 3(b) describes how data from photovoltaic (PV) panels is collected. This data includes metrics for power generation, panel temperature, and sunlight irradiance. It is essential for optimizing solar power output through the manipulation of panel orientations and the control of energy conversion efficiency. The colored lines show how data is sent directly and continuously to the control center, allowing for operational modifications and real-time analysis. The focus of Fig. 3(c) is performance monitoring and predictive maintenance for wind and solar systems. It demonstrates how temperature monitors on solar inverters and vibration sensors on wind turbines gather data at preset intervals or in response to particular triggers. These less common but highly targeted data collecting patterns represented by unique turquoise colored line seeks to detect early indicators of wear, possible breakdowns, or inefficiencies in order to facilitate prompt maintenance measures and minimize unscheduled downtime.

Fig. 3(d) shows the recorded information on battery charge levels, input/output power, and the general health and efficiency of storage units, demonstrates the crucial function that energy storage devices play within the smart grid. Given the erratic nature of wind and solar power output, the data flow that is being highlighted here is crucial for maintaining a balance between the supply and demand of energy. The information is strategically routed as indicated by the unique black colorful line, guaranteeing that energy storage is managed as efficiently as possible to maintain grid stability and dependability. Fig. 3(e), which provides a broad overview of the data integration and flow from all wind and solar powered systems, such as storage, wind, and solar power, to the smart grid control center. The intricate, multi-domain data gathering tactics are highlighted in this figure, which also shows how operational, maintenance, and performance data are combined to provide information for demand response and real-time grid management. The black colored lines highlight how the smart grid can adaptively control energy production, storage, and distribution in response to changing demands and conditions. They also show how harmonized data flow occurs across various time scales and frequencies.

Figs. 3(f) to (g) illustrate the consequences of DoS attacks aimed to expose vulnerabilities in the distributed energy and power systems. Initially, the data transmission between energy systems proceeds normally for the first few seconds. However, as time progresses, the integrity of the received data deteriorates; it becomes increasingly difficult to discern the values within the timeframe of 0.081 seconds to 0.3 seconds due to manipulation. Subsequently, the data signals return to normalcy as the blockchain algorithm initiates an immediate recovery process after receiving input from the hybrid machine learning algorithm for the compromised nodes, effectively isolating them in the network. The similar process is repeated in different time domain cycles in other figures. However, the impact of DDoS attacks is observed to be more severe on the DERs in the SG, as illustrated from Fig. 3(h) to (n). Initially, there is a noticeable deviation in the data transmission performance among different energy systems. The integrity of the received data significantly deteriorates, making it challenging to accurately discern values within the timeframe of 0.081 seconds to 0.3 seconds due to manipulation, as demonstrated in Fig. 3(h).

Subsequently, normalcy in data signals is restored as the blockchain algorithm initiates an immediate recovery process, reinforced by insights from a hybrid machine learning algorithm, for the compromised nodes, thereby effectively isolating them in the network. This recovery mechanism is consistently applied in various time domains in all subsequent figures. In both cases, the proportion of compromised nodes were remained below 51%, enabling the blockchain algorithm, with the assistance of the hybrid machine learning algorithm, to commence the recovery of the compromised nodes in the DERs. Finally, the most severe scenario, combining both DoS and DDoS attacks, is depicted in Fig. 3(o), where the entire data packets signals are corrupted when received at the control center. In this case, it becomes significantly challenging for the blockchain algorithm to facilitate recovery in a short time as observed in previous figures, though machine learning algorithm provides identifications of the cyberattacks. The primary reason for slow recovery is the escalation in the number of compromised nodes beyond the 51% threshold level, complicating the system's ability to autonomously recover from the DoS and DDoS cyberattacks in the smart grid.

## 4 Experimental Design, Materials, and Methods

**Fig. 4:**
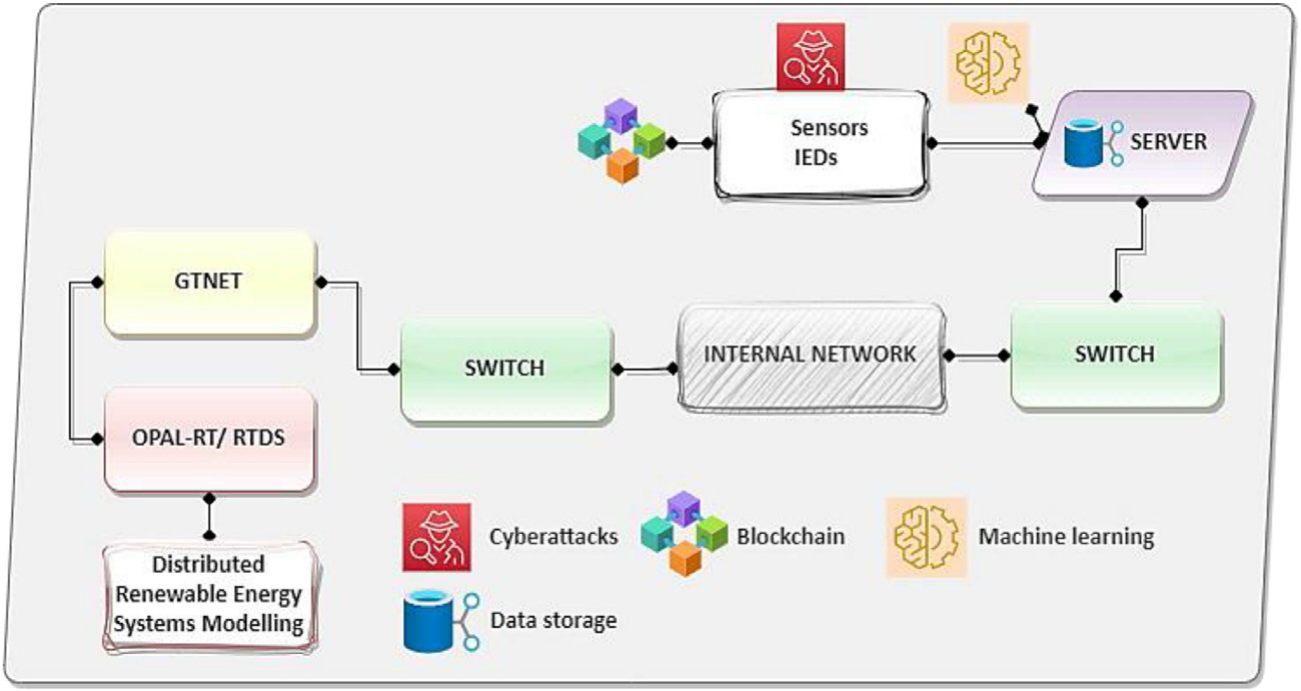
Simulation design and testing in smart grid.

The simulation model is depicted in Fig. 4 consists of a network that incorporates several sensor nodes in the smart grid. These nodes are crucial for gathering data from multiple energy and power systems in the smart grid. The data collected by nodes is transmitted to a centralized data storage server, which serves as the core for information management and aggregation. The nodes are connected to the data storage server over a blockchain-based wireless network architecture, offering continuous data transfer to the central repository [21,22]. In a hybrid topology, nodes sense real-time measurements including voltage, current, signal strength, network traffic, and bandwidth usage, along with power parameters. This setup allows for continuous monitoring of the energy and power systems status. Furthermore, the nodes collect information on the status of critical equipment and generate event reports that detail system events and faults. They also enable the timely transmission of control commands for device management. Additionally, the nodes gather security metrics to guarantee the integrity of data transmission and evaluate the connectivity status of devices across both wired and wireless networks. In sum, the data gathered by nodes enable a comprehensive understanding of the smart grid, enhancing the monitoring, control, and optimization of its components. Consequently, by employing advanced analytical methods, like machine learning (as explained in Data Description Section), identify patterns and anomalies in the data impacted by DoS and DDoS cyberattacks. This enables the early detection of potential security breaches or unauthorized access attempts in power and energy systems. This setup not only ensures the integrity and reliability of the data collected but also supports efficient and secure data handling. Moreover, the data storage server is directly linked to the RTDS, a tool vital for simulating real-time operations. The connection, established over an internal network, underscores the effective collaboration between the simulation tool and the data storage system. The RTDS is essential for modeling power and energy sources, such as wind turbines and solar panels models in the smart grid.

The administrator has access to all of this updated information, allowing to take timely and crucial actions to mitigate cybersecurity threats in energy and power systems. In this manner, it lays the groundwork for a reliable, resilient, and secure energy infrastructure that benefits consumers, businesses, and society at large. In addition, the blockchain architecture is simulated with the help of programming tools C++, Java, and Rust installed on a virtual computer running Fedora32. The path loss model [23], synchronization between nodes [24], and positioning technique [25,26] were employed to identify the energy systems and nodes location in the SG. In this study following simulation parameters have been used to perform simulations. Simulation parameters and their values used in this study are given in Table 3.

**Table 3:** Simulation parameters and values.

Simulation Parameters	Values
Simulator	RTDS
Blockchain architecture	Distributed
Communication technology	5G and Ethernet
Frequencies in coverage	400MHz-450MHz/ 700MHz
Firewall	Checkpoint
Switches	Layer 1 and 2 (configurable)
Transmission and control standards	IEC 60870-5-104, IEC 60870-5-101
Wireless sensors	200 (MICAz, TelosB)
Physical layer IEEE and IEC Standard	IEEE 802.15.4, 61850 (Goose)
Grid Power	300Megawatts
Wind turbines	15
Roto Blade radius	41m
Height above ground	80m
Initial sensor node energy	15J
Wind Speed (cu-in, nominal, cut-out)	3.5, 13, 2o m/s 12.6, 46.8, 72 km/hr
Nominal turbine speed	14.4 rpm
Induction machine speed at rated power	1214 rpm
Induction machine	6poles, 1200rpm
Gear box ratio	84.5
Monocrystalline solar panels efficiency	15.5-18%
Solar panels	50
heated solar panel from	35°C to –50°C
Packet receiving power	0.05W
Idle listening	0.021W
Sleeping power	0.0015W
Data aggregation	0.013W
Packet length	79 bytes
Wireless data transfer rate	256 kbps
Maximum hop distance	7m
Gas value	0.00015
Buffer size	10Mb
Data transmission rate	300Mbps
Path loss for LoS and non-LoS	-91 to -93
Noise floor for LoS and non-LoS	-89, -97
Shadowing deviation for LoS and non-LoS	1.01, 1.22
Area: 2D (length×width)	1000m × 1000m
Simulation time	150 sec
Set of simulations	30

## Limitations

The computational complexity of hybrid machine learning algorithm in blockchain-based networks can introduce latency in real-time detection and response systems, potentially delaying the mitigation of such stealthy cyberattacks. This limitation underscores the challenge in balancing between detection accuracy and the need for prompt response in the dynamic environment of the smart grid applications.

## Ethics Statement

The data presented in this study did not involve using human or animal subjects or social media platforms, or stealing other people's data. Consequently, no ethical statements as per the journal policy were required for the data.

## CRediT Author Statement

**Muhammad Faheem**: Writing original draft, Conceptualization, Methodology, Software, Testing and Validation, Review and Editing. **Mahmoud Ahmad Al-Khasawneh**: Data Validation, Data Curation.

## Data Availability

Multilayer Cyberattacks Identification and Classification Using Machine Learning in Internet of Blockchain (IoBC)-Based Energy Networks. (Original data) (Mendeley Data).
